# mtDNA mutations, hearing loss and aminoglycoside treatment in Mexicans

**DOI:** 10.1590/S1808-86942011000500006

**Published:** 2015-10-22

**Authors:** Meza G, Torres-Ruíz NM, Tirado-Gutiérrez C, Aguilera P

**Affiliations:** 1 Ph.D (Researcher); 2 Ph.D (Researcher); 3 Ph.D (Researcher); 4 Ph.D (Researcher)

**Keywords:** aminoglycosides, dna, mitochondrial, genes, hearing loss, rna

## Abstract

**Abstract:**

Streptomycin and aminoglycoside derivatives are commonly used to treat tuberculosis and other stubborn infections; these drugs may alter auditory and/or vestibular function. Mutations in mitochondrial DNA have been associated with hypersensitivity to aminoglycosides; no studies have been conducted in Mexicans, which are very prone to such alterations because aminoglycosides have been prescribed carelessly for many years, irrespective of the ailment to be treated.

**Aim:**

We investigated “hot spot” mutations described previously as causing inner ear alterations.

**Methods:**

Hot spot mutations at the 12S rRNA gene and the tRNA Serine (UCN) gene were screened by PCR-RFLP and sequencing in 65 subjects undergoing audiological and vestibular testing.

**Study Design:**

Experimental.

**Results:**

32 individuals had healthy auditory and vestibular function, whereas 33 subjects had auditory affections. We found none of the previously reported mutations related to aminoglycoside hypersensitivity, or non-syndromic hearing loss. Two hearing-impaired patients that had been treated with streptomycin had the T1189C variant of the mitochondrial 12S rRNA region.

**Conclusion:**

Mutations related to hearing loss in other ethnic backgrounds were not found in Mexicans. However, the T1189C variant is possibly a putative mutation related to aminoglycoside hypersensitivity and was present in 2 patients.

## INTRODUCTION

Deafness is one of the main diseases among the human world population. In Mexico, 5 of every 1,000 neonates are born deaf, contrasting with 1 of 1,000 deaf newborn children worldwide^1^. Among the former, 25% are genetically determined, 25% have a non-genetic origin, and the etiology is unknown in the remaining 50%. Deafness may be syndromic when one or more systems besides the auditory apparatus are affected; in non-syndromic hypoacusia, only the auditory system is affected (pure deafness). Non-syndromic hypoacusia is frequently inherited from the mother, indicating that genetic changes occur in mitochondrial DNA (mtDNA). Inner ear structures are very sensitive to injury because of their specific physiologic energy requirements (ion carriers and channels)^2^. A silent sudden and dangerous cause of non-syndromic deafness occurs when mtDNA confers a high sensitivity to aminoglycosides (streptomycin family); these drugs are often used in the treatment of stubborn infections, such as Tuberculosis, which do not respond to others antibiotics^3^.

Aminoglycosides affect mainly molecules involved in protein synthesis; they inhibit protein synthesis initiation by interacting with the site where the anticodon tRNA region (usually tRNA methionine) meets the A site of ribosomal RNA. This mechanism has been elucidated in bacterial 16S rRNA, and probably occurs in mitochondrial 12S rRNA^4^,^5^.

The non-syndromic deafness-associated 12S rRNA region has been studied in several ethnic groups, and putative mutations have been described^6^,^7^, namely: T961insC, T961C and T961+C(n)ins in Chinese patients^8^ and Italian families^9^; T1095C and C1494T in Chinese^10^, Spanish^11^ patients and Spanish families^12^; and A1555G in Korean patients^13^ and Japanese^14^, Chinese^15^, and Italian families^16^. The tRNA Serine^(UCN)^ A7445G mutation gene has also been related with non-syndromic-deafness^17^ has also been investigated.

Mitochondrial DNA is transmitted by the mother; its polymorphisms (also named “variants”), when present in certain sites, seem to increase the risk or susceptibility to develop certain neurodegenerative conditions such as hearing loss^18^.

Because aminoglycoside are used frequently (it is the treatment of choice for tuberculosis), we decided to investigate three different changes or hot spot mutations at the 3' end of the 12S rRNA region (T961+C(n)ins, A1555G, and C1494T), and tRNA Serine^(UCN)^ (A7445G) of mitochondrial DNA that could be associated with aminoglycoside hypersensitivity in patients previously screened for healthy or compromised auditory and/or vestibular function.

## MATERIALS AND METHODS

### Subjects

The study sample comprised 65 Mexican subjects selected at the Instituto Nacional de Enfermedades Respiratorias, Mexico, who agreed to participate (Protocol B04-02). The clinical history was taken, focusing on past aminoglycoside treatments. Vestibular and auditory function was investigated in all selected individuals Peripheral blood samples were taken for DNA extraction (DNAzol-Invitrogen, technique provided by the suppliers) and screened for the presence of hot spot mitochondrial DNA mutations related to non-syndromic-deafness.

### Audiological and vestibular evaluation

The age of individuals ranged from 5 to 70 years (mean - 35 years). Otoscopy and pure tone audiometry at thresholds of 25, 250, 500, 1000, 2000, 4000, and 8000 Hz (Interacustic AC 40 audiometer) were carried out. Hearing above 25 dB was considered normal; hearing loss was mild (25 to 40 dB), moderate (45 to 60 dB), severe (65 to 80 dB), and profound (85 to 120 dB). Caloric tests were also run in all individuals to assess vestibular damage.

### Polymerase chain reaction-restriction fragment length polymorphism (PCR-RFLP)

The technique was performed as in Hutchin T, et al for A1555G^19^ for 961 site mutations (324-349 and 1282-1307, *Mnl)*, and A7445G (7399-7419 and 7751-7771, *Xba I*). The procedure consisted of denaturation at 95°C for 5 min, followed by 35 cycles (95°C for 20s, 50°C for 30 s, and 72°C for 30 s), and a final extension at 72°C for 7 min. Each PCR fragment was digested by enzymatic restriction with the abovementioned enzymes and separated by electrophoresis in agarose 3%.

For the C1494T mutation and other variants around the A1555G region, PCR amplification was done using the Phusion DNA polymerase (New England Biolabs) and oligonucleotides corresponding to the positions 1011-1134 and 1624-1644. The PCR conditions were: denaturation at 98°C for 5 min, followed by 35 cycles (98°C for 30s, 60°C for 30 s, and 72°C for 45s), and a final extension at 72°C for 5 min. Each fragment was column purified (Quiagen) and each sample was subsequently analyzed twice by direct bidirectional sequencing in an ABI PRISM 310 Genetic Analyzer Perkin-Elmer Applied Biosystems. The sequence alignment was performed with BLAST and DNAstar programs and compared with the consensus Cambridge sequence (GenBank Accession No. NC_012920), Human Mitochondrial Genome Database. Chromatograms were all manually checked for sequence analysis.

The sequences of the following species were used for alignment to search for conservation of the sites where the variants were located in the 12S rRNA gene: human (NC_012920), cow (NC_006853), dog (NC_002008), rat (NC_001665), chimp (NC_001643), gorilla (NC_001645) and mouse (NC_005089).

## RESULTS

**Clinical findings**

**Figure 1:**
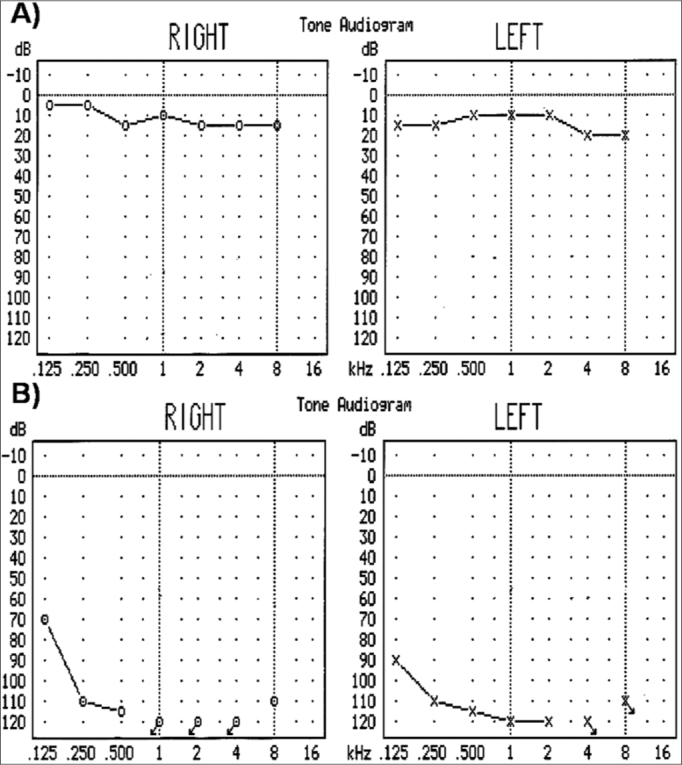
Audiograms - analysis of the 12S rRNA gene - Audiograms of subjects analyzed in the 12S rRNA gene. (A) Healthy individual without audiometric alterations and (B) Patient with hearing loss.

Among the 65 study subjects, audiological and vestibular evaluation revealed that 32 had healthy auditory and vestibular function, whereas the remaining 33 presented hearing loss (Figure 1). Of these latter, 18 patients had profound hearing loss, 14 had mild hearing loss, and only 13 had vestibular damage. Aminoglycoside therapy had been given to 15 subjects; 18 subjects were not treated.

### PCR-RFLP and sequences analysis

When the reported sites (961 insCn, C1494T, A1555G, and A7445G) were screened, they were not found in any of the samples of the study groups. However, we found a change (thymidine to cytosine) at the 1189 site in the 12S rRNA gene of 2 patients with auditory and vestibular diseases, who had been treated with streptomycin (one of them had been treated additionally with kanamicyn).

## DISCUSSION

The 12S rRNA region in mitochondrial DNA has been described as a hot spot for nucleotide variability related with non-syndromic deafness as a result of aminoglycoside hypersensitivity; several reports have confirmed this finding. In our molecular biological studies, we found no mutation associated with hearing loss and aminoglycoside hypersensitivity.

**Figure 2:**
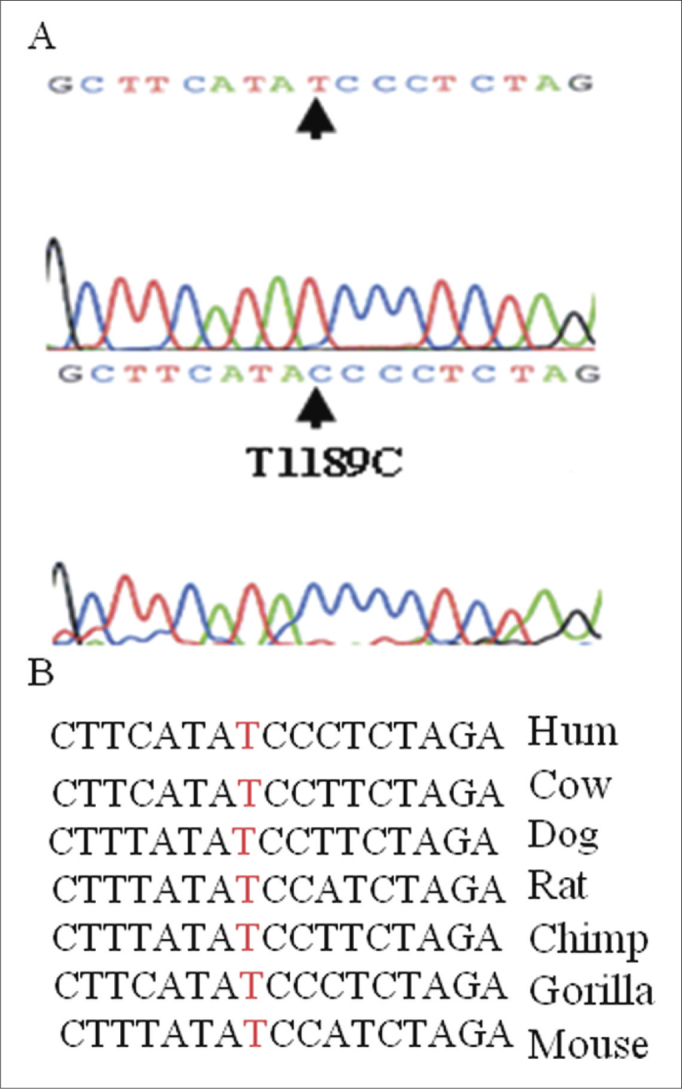
T1189C in 12S rRNA - (A) Partial chromatogram sequences of the 12S rRNA gene in T1189C carriers, and (B) Partial sequence alignment of the 12S rRNA gene in different species showing that the position 1189 is highly conserved.

However, we found a T to C change in the position 1189 in two patients, irrespective of alignment with the universal Cambridge database, which is the first report in the literature (to our knowledge) of such a nucleotide sequence change in the region close to the 3' end of the mitochondrial 12S rRNA gene, which could be correlated with auditory or vestibular alterations due to aminoglycosides (Figure 2A).

Analysis of the position 1189 in different species has shown that it is a highly conserved site (Figure 2B). Whether or not it is a result of aminoglycoside hypersensitivity in patient would have to be investigated in a larger series. The T1189C variant is in the region where C1494T and A1555G changes have been found; their action may be attributed to closeness to the ribosome site A where protein synthesis is initiated.

## CONCLUSION

Our results suggest that mutations associated with hearing loss and aminoglycoside toxicity, which are found in other ethnic groups, were not present in Mexicans. Furthermore, the T1189C variant, which had not been previously reported, is apparently associated with aminoglycoside treatment. Whether this putative mutation is present in other individuals is of significant importance, as it may be a valuable screening marker prior to therapy. Recommendations may be given to avoid damage due to these hazardous antibiotics.

## ACKNOWLEDGMENTS

The authors wish to thank Doctor Robyn Hudson, of the Biomedical Institute (UNAM), for a critical review and English grammar corrections in the manuscript; also Doctor Ignacio Mora Magaña for some of the audiological measurements, and Mrs Cecilia Escalona Ochoa for much help in the laboratory. We thank Doctor Daniel Ortuño Sahagún for providing access to the reverse alignment DNAstar program, and the technical personnel of the Unit of Molecular Biology for significant support.
